# Anatomical organization of the lateral cervical nucleus in Artiodactyls

**DOI:** 10.1007/s11259-021-09788-1

**Published:** 2021-04-17

**Authors:** Annamaria Grandis, Anna Gardini, Claudio Tagliavia, Giulia Salamanca, Jean-Marie Graïc, Margherita De Silva, Cristiano Bombardi

**Affiliations:** 1 Department of Veterinary Medical Sciences, University of Bologna, Ozzano dell’Emilia, BO Italy; 2 Department of Comparative Biomedicine and Food Science, University of Padova, Legnaro, PD Italy

**Keywords:** Lateral cervical nucleus, Calbindin-D28k, Neuronal nitric oxide synthase, Calf, Pig

## Abstract

The presence of the lateral cervical nucleus (LCN) in different mammals, including humans, has been established in a number of anatomical research works. The LCN receives its afferent inputs from the spinocervical tract, and conveys this somatosensory information to the various brain areas, especially the thalamus. In the present study, the organization of the calf and pig LCN was examined through the use of thionine staining and immunohistochemical methods combined with morphometrical analyses. Specifically, the localization of calbindin-D28k (CB-D28k) and neuronal nitric oxide synthase (nNOS) in the LCN was investigated using the immunoperoxidase method. Calf and pig LCN appear as a clearly defined column of gray matter located in the three cranial segments of the cervical spinal cord. Thionine staining shows that polygonal neurons represent the main cell type in both species. The calf and pig LCN contained CB-D28k-immunoreactive (IR) neurons of varying sizes. Large neurons are probably involved in the generation of the cervicothalamic pathway. Small CB-D28k-IR neurons, on the other hand, could act as local interneurons. The immunoreactivity for nNOS was found to be mainly located in thin neuronal processes that could represent the terminal axonal portion of nNOS-IR found in laminae III e IV. This evidence suggests that nitric oxide (NO) could modulate the synaptic activity of the glutamatergic spinocervical tracts. These findings suggest that the LCN of Artiodactyls might play an important role in the transmission of somatosensory information from the spinal cord to the higher centers of the brain.

## Introduction

The lateral cervical nucleus (LCN) is a column of gray matter located in the most caudal part of the medulla and in the dorsal portion of the lateral funiculus of the C1, C2, and C3 spinal segments (Rexed and Brodal 1951). This nucleus receives mechanical and noxious stimuli from the skin (including hair) through the spinocervical tract, which originates in laminae III and IV along the length of the ipsilateral spinal cord (Cervero et al. 1977; Craig and Tapper 1978; Brown et al. 1980; Craig et al. 1992; Pubols and Haring 1995), and primarily projects to the contralateral ventroposterior and posterior regions of the thalamus (Smith and Apkarian 1991; Broman and Ottersen 1992; Zhang and Broman 1998). Glutamate is the primary excitatory neurotransmitter of the somatosensory and nociceptive systems (Bhave et al. 2001). However, many physiological neuronal processes, such as signal transduction, are also regulated by other neuroactive molecules, such as calbindin-D28k (CB-D28k) and the neuronal nitric oxide synthase (nNOS), as it is in other areas of the Artiodactyl brain (Bombardi et al. 2006; Pirone et al. 2021). CB-D28k and nNOS are localized within neurons and neuropilar areas widely distributed in the dorsal, lateral and ventral horns of the spinal cord. In particular, the localization of a large amount of CB-D28k- and nNOS-immunoreactive (IR) neurons in the superficial layers of the dorsal horn suggests that these proteins play a role in sensory and nociceptive functions (Nazli and Morris 2000). Despite numerous studies conducted on the LCN of different mammals, only one anatomical study has been published over 50 years ago regarding someanatomical features of the LCN of Artiodactyls (*Artiodactyla*) (Verhaart 1970). It must be noted that the latest studies on the anatomical organization of the LCN in the different species date back to about twenty years ago. Furthermore, ruminants and suidae have large convoluted brains characterized by high level structures that can process information related to pain signals (Ballarin et al., 2016; Minervini et al. 2016; Graïc et al. 2018; Pirone et al. 2018, 2019; Corain et. al 2020; Peruffo et al. 2019; Cozzi et al. 2020). Therefore, in the present study, thionine staining combined with morphometrical analyses were used to determine the previously uncharted cytoarchitectonic characteristics of the LCN in calves (*Bos taurus*) and pigs (*Sus scrofa*). In addition, the distribution of CB-D28k and nNOS immunoreactivity in the LCN was determined using the immunoperoxidase technique.

## Materials and methods

### Animals and fixation

The tissues employed for this study were obtained from three calves (male, friesian, body weight 312 ± 6 kg,) and three pigs (male, large white, body weight 168 ± 5 kg) aged 10 months. These animals were euthanized due to extra-neurological disorders (overall different pathologies concerning paratopias of the digestive system). All procedures involving animals were carried out in accordance with the Italian legislation regarding experimental animals, after approval by the Ethic Scientific Committee for Experiments on Animals of the University of Bologna (Prot Rif. BQ/gf PROT 13,825–X/10 – All. 67). The animals were deeply anesthetized and sacrificed through the administration of embutramide, mebenzonium iodide and tetracaine hydrochloride (Tanax). The upper spinal cord (C1, C2 and C3 spinal segments) of all the utilized subjects was immediately removed and fixed in 4% paraformaldehyde in 0.1 M sodium phosphate buffer at 4 C° for 48 h. The samples were washed in a phosphate buffer saline (PBS; 0.15 M NaCl in 0.01 M sodium phosphate buffer, pH 7.2) solution, and later stored in a PBS solution containing 30% sucrose and 0.1% sodium azide (pH 7.4) at + 4 C°. Coronal sections were cut at a thickness of 40 µm using a freezing sliding microtome. The sections were stored in a PBS solution containing 30% sucrose and 0.1% sodium azide (for immunohistochemical staining) or in 4% paraformaldehyde (for thionine staining) at + 4° C until further processing.

### Immunoperoxidase experiments

The free-floating coronal sections were collected from the PBS solution containing 30% sucrose and 0.1% sodium azide, and washed in 0.01 M PBS for 3 times. To eliminate endogenous peroxidase activity, the sections were treated with 1% H2O2 in PBS for 15 min, and then washed in PBS six times. To block non-specific binding, the sections were incubated in a solution containing 10% normal goat serum (Sigma-Aldrich, G9023, Missouri, USA) and 0.5% Triton X-100 in PBS at room temperature (RT) for 2 h. Thereafter, the sections were soaked in a solution containing a rabbit anti-calbindin D-28 k polyclonal antibody (diluted 1:1000; CB-38a, Swant, Marly, Switzerland) or a mouse anti-nNOS monoclonal antibody (diluted 1:80, sc-5302, Santa Cruz Biotechology, CA, USA) at 4 °C for 48 h. The final concentrations of the primary antibodies were established by performing immunoperoxidase reactions using different dilution patterns. The primary antibodies were diluted in an antibody diluent (1.8% NaCl in 0.01 M sodium phosphate buffer containing 0.1% sodium azide) containing 1% normal goat serum and 0.5% Triton X-100. Afterwards, the sections were washed in PBS 3 times (10 min each). Thereafter, the sections were incubated in a solution containing a goat biotinylated anti-rabbit antibody (1:200, BA-1000, Vector Laboratories, Burlingame, CA, USA) or a goat biotinylated anti-mouse antibody (diluted 1:200, BA-9200, Vector Laboratories, Burlingame, CA, USA), 1% normal goat serum, and 0.3% Triton X-100 in PBS at RT for 2 h. Subsequently, the sections were washed in PBS 3 times for 10 min each and were then soaked in avidin–biotin complex (ABC kit Vectastain, PK-6100, Vector Laboratories, Burlingame, CA, USA) at RT for 45 min. After another three washes in PBS, the immunoperoxidase reaction was developed in a solution containing 3–3′-diaminobenzidine (DAB kit, SK-4100, Vector Laboratories, Burlingame, CA, USA). Finally, the slides were dried overnight, dehydrated in ethanol, cleared in xylene, and coverslipped with Entellan (Merck, Darmstaldt, Germany).

### Antibody specificity and controls

Using the immunoperoxidase method, no detectable immunolabeling was evidenced on the control sections, for which the primary antibodies had been omitted. The omission of the primary antibodies, as well as the replacement of the secondary antibodies with unsuited secondary antibodies resulted in the elimination of all immunohistochemical staining. In every batch, a positive control was included to ascertain the specific staining.

### Thionine staining

To determine the anatomical boundaries and the cytoarchitectural features of the LCN, the sections that were adjacent to the immunoperoxidase preparations were stained with thionine, as described hereafter. Sections were removed from the 4% paraformaldehyde solution, mounted on gelatin-coated slides, and dried at 37° C overnight. Afterwards, the sections were defatted in a mixture of chloroform/ethanol 100% (1:1) for 1 h, and then rehydrated through a progressive series of decreasing concentrations of ethanol (3 min in 100% ethanol, 2 min in 95% ethanol, 2 min in 90% ethanol, 2 min in 80% ethanol, 2 min in 70% ethanol, 2 min in 50% ethanol), immersed in dH2O for 2 min, and finally stained in a 0.125% thionine (Fisher Scientific) solution for 15 min, dehydrated and coverslipped with Entellan (Merck, Darmstaldt, Germany).

### Analysis of sections

The sections, stained with thionine and with immuneperoxidase procedures, were observed with a Zeiss Axioplan microscope (Carl Zeiss, Oberkochen, Germany). A KS 300 Zeiss software (Kontron Elektronik, Germany) was used for the morphometric analysis of thionine-stained and immunostained neurons. Only the neurons showing an evident nucleus (as well as a nucleolus, in the thionine-stained sections) were counted and included in the perikaryal area analysis. These data, obtained using twelve nonconsecutive sections from each cervical segment (of each animal), were measured after having manually traced the cell body outlines. Data were expressed as mean ± standard deviation (SD). The contrast and brightness of the figures were adjusted to resemble the appearance of the labelling seen through the microscope using Adobe Photoshop CS3 Extended 10.0 software (Adobe Systems, San Jose, CA).

## Results

### Bovine lateral cervical nucleus: thionine staining

**Fig. 1:**
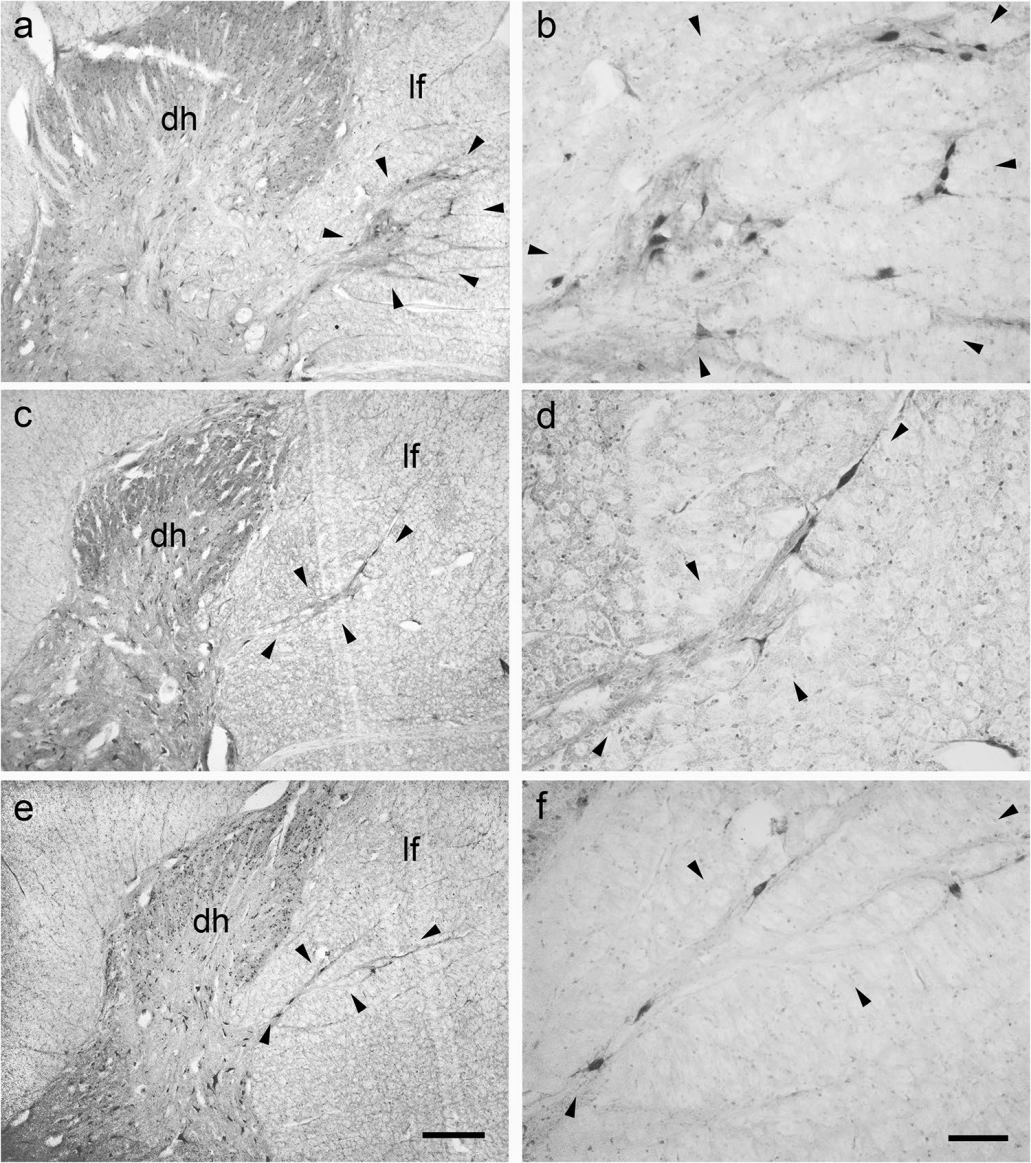
Brightfield photomicrographs of thionine-stained coronal sections showing the topography of the bovine lateral cervical nucleus (bordered by arrowheads). The three sections are arranged from cranial to caudal direction: C1 (**a**-**b**), C2 (**c**-**d**) and C3 (**e**–**f**). Abbreviations: dh, dorsal horn; lf, lateral funiculus. Scale bar = 400 µm in e (applies to **a**, **c**, **e**) and 100 µm in **f** (applies to **b**, **d**, **f**)

**Fig. 2:**
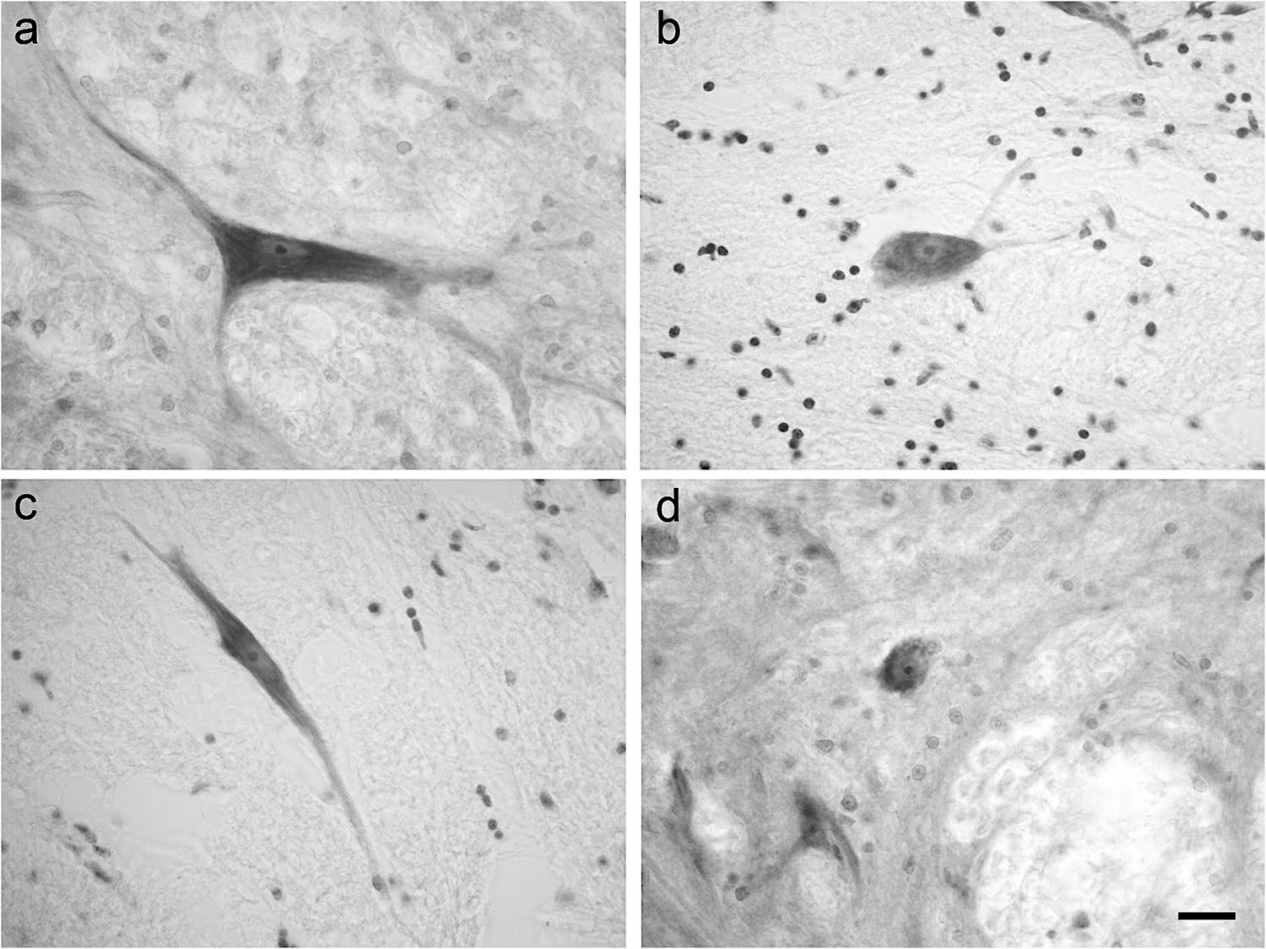
Brightfield photomicrographs of thionine-stained transverse sections showing the morphology of the bovine lateral cervical nucleus neurons: polygonal (**a**, **b**), fusiform (**c**) and spheroidal (**d**). Scale bar = 20 µm in d (applies to **a**-**d**)

A well-developed LCN was present in the dorsal part of the bovine lateral funiculus. This nucleus appeared as a longitudinal column of neurons extending from the cranial part of C1 to the caudal end of C3 (Fig. 1a-f). The calf LCN is large and elongated in transversal plane and often connected to the dorsal horn or the intermediate zone of the spinal gray matter. The LCN shifts from lateral to ventrolateral to the neck of the dorsal horn (Fig. 1a-f). A total of 561 clearly visible neurons was counted in the calf LCN. Most of the LCN neurons (54%) were located in the C1 spinal segment (303 C1 neurons / 561 LCN neurons). C2 and C3 contained 22.1% (124 C2 neurons /561 LCN neurons), and 23.9% (134 C3 neurons /561 LCN neurons) of neurons, respectively. The calf LCN neuronal populations could be divided into three morphological cell types: polygonal, fusiform, and spheroidal (Fig. 2a-d). Polygonal neurons were multipolar with angular soma from which a minimum of three primary dendrites of variable thickness arose (Fig. 2a, b). The somata of fusiform cells had an ovoidal shape with primary dendrites originating from the opposite poles of the soma (Fig. 2c). Spheroidal cells had a round cell body and did not show any visible dendrite (Fig. 2d). Spheroidal neurons were not observed at C3 level. The distribution and the perikaryal area of the different neuronal types in the bovine LCN are reported in Tables 1, 2, 3, 4 and 5. We did not observe a clear division of the bovine LCN. However, the dorsolateral neurons appeared larger than the ventromedial ones.

**Table 1:** Distribution of polygonal, fusiform and spheroidal neurons in bovine lateral cervical nucleus

Spinal segments	Polygonal neurons	Fusiform neurons	Spheroidal neurons	Total neurons counted
C1	235 (77.5%)	46 (15.2%)	22 (7.3%)	303
C2	111 (89.5%)	10 (8.1%)	3 (2.4%)	124
C3	129 (96.3%)	5 (3.7%)	0 (0%)	134
Total C1-C3	475 (84.7%)	61 (10.9%)	25 (4.4%)	561

**Table 2:** Bovine lateral cervical nucleus: perikaryal area (µm^2^) of total neuronal population

Spinal segments	Mean area ± standard deviation	Minimum area	Maximum area
C1	358.5 ± 210.3	33.4	1408.1
C2	270.4 ± 129.4	45.1	591.4
C3	252.7 ± 135,5	53.9	1024
Total C1-C3	313.8 ± 185.5	33.4	1408.1

**Table 3:** Bovine lateral cervical nucleus: perikaryal area (µm^2^) of polygonal neurons

Spinal segments	Mean area ± standard deviation	Minimum area	Maximum area
C1	376.8 ± 220.3	33.4	1408.1
C2	268.1 ± 131.3	45.1	591.4
C3	250.9 ± 136.2	53.9	1024
Total C1-C3	317.2 ± 191	33.4	1408.1

**Table 4:** Bovine lateral cervical nucleus: perikaryal area (µm^2^) of fusiform neurons

Spinal segments	Mean area ± standard deviation	Minimum area	Maximum area
C1	296.7 ± 163.6	91.1	872.5
C2	283.7 ± 110.4	148	436.8
C3	299.1 ± 116.6	137.2	391.7
Total C1-C3	296.1 ± 151.8	91.1	872.5

**Table 5:** Bovine lateral cervical nucleus: perikaryal area (µm^2^) of spheroidal neurons

Spinal segments	Mean area ± standard deviation	Minimum area	Maximum area
C1	287 ± 160.2	83.9	771.7
C2	312.3 ± 154	178.7	480.8
C3	/	/	/
Total C1-C3	290.1 ± 156.4	83.9	771.7

### Porcine lateral cervical nucleus: thionine staining

**Fig. 3:**
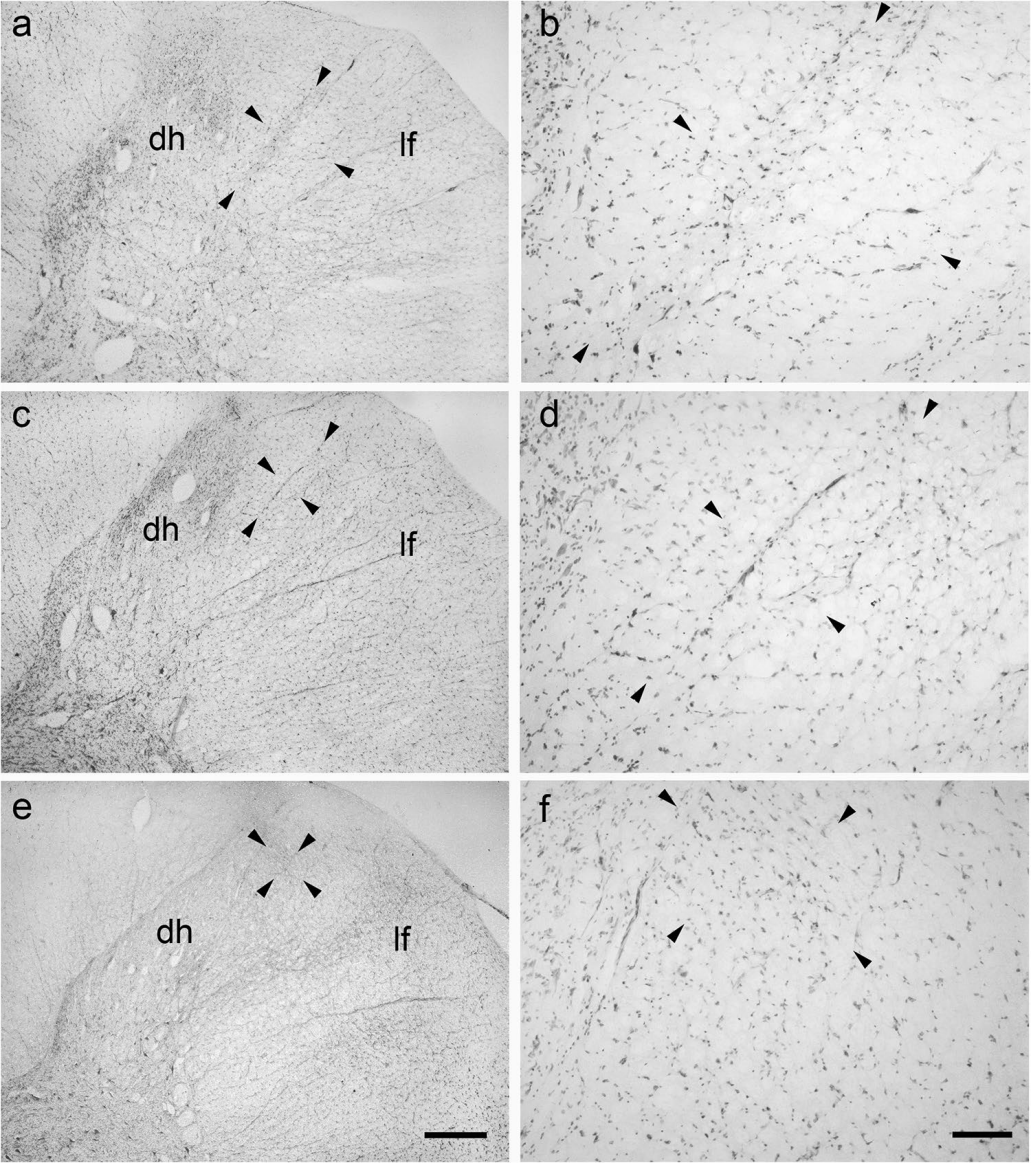
Brightfield photomicrographs of thionine-stained coronal sections showing the topography of the porcine lateral cervical nucleus (bordered by arrowheads). The three sections are arranged from cranial to caudal direction: C1 (**a**-**b**), C2 (**c**-**d**) and C3 (**e**–**f**). Abbreviations: dh, dorsal horn; lf, lateral funiculus. Scale bar = 400 µm in e (applies to **a**, **c**, **e**) and 100 µm in f (applies to **b**, **d**, **f**)

**Fig. 4:**
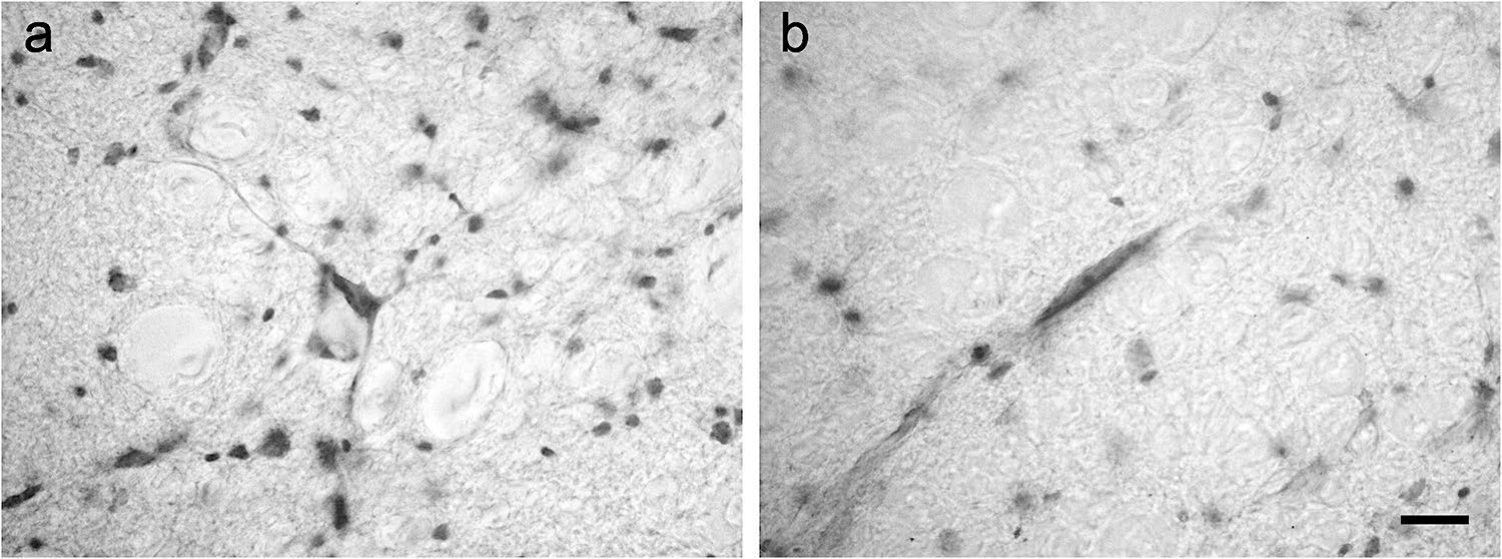
Brightfield photomicrographs of thionine-stained transverse sections showing the morphology the of the porcine lateral cervical nucleus neurons: polygonal (**a**) and, occasionally, fusiform (**b**). Scale bar = 20 µm in b (applies to **a**, **b**)

The pig LCN consisted of a longitudinally-oriented column of neurons which was less evident than that seen in calves. This small group of neurons was located in the dorsal portion of the lateral funiculus of the three cranial cervical spinal segments (Fig. 3a-f). As found in the calf, the LCN changes its position from lateral to ventrolateral to the dorsal horn (Fig. 3a-f). The connections between the LCN and the spinal gray matter were less consistent than those observed in calves. All porcine LCN neurons showed a polygonal shape (Fig. 4a), whereas ovoidal neurons were not evidenced. Few fusiform neurons were occasionally observed (Fig. 4b). In the pig LCN, a total of 189 clearly-visible neurons were counted in thionine preparations. As with the calf, the number of neurons decreased cranio-caudally: C1 contained (81/189 LCN neurons) 42.8%, C2 (51/189 LCN neurons) 27% and C3 (57/189 LCN neurons) 30.2% of neurons. The perikaryal areas of the neuronal population in the pig LCN are reported in Table 6.

**Table 6:** Porcine lateral cervical nucleus: perikaryal area (µm^2^) of total neuronal population

Spinal segments	Mean area ± standard deviation	Minimum area	Maximum area
C1	183.1 ± 88.4	42.8	486.8
C2	169.2 ± 97.5	44	516.9
C3	192.3 ± 114.1	70.8	658.4
Total C1-C3	182.1 ± 99	42.8	658.4

### Bovine lateral cervical nucleus: calbindin-D28k immunoreactive neurons

**Fig. 5:**
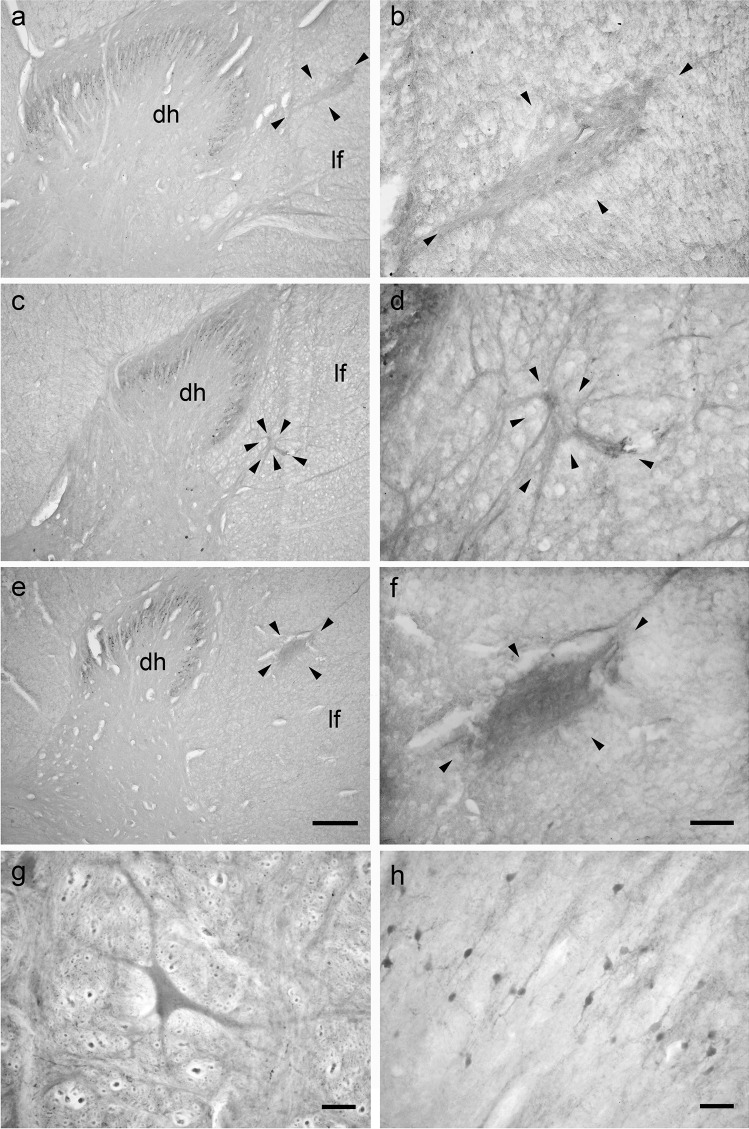
Brightfield photomicrographs of transverse sections showing the distribution of calbindin-D28K (CB-D28k) immunoreactivity in the bovine lateral cervical nucleus (bordered by arrowheads). The three sections are arranged from cranial to caudal direction: C1 (**a**-**b**), C2 (**c**-**d**) and C3 (**e**–**f**). Note that CB-IR neurons have polygonal somata from which evident primary dendrites arise (**g**). Generally, these neurons are less intensely immunostained than those located in superficial layers of the dorsal horn (**h**). Abbreviations: dh, dorsal horn; lf, lateral funiculus. Scale bar = 400 µm in e (applies to **a**, **c**, **e**), 100 µm in **f** (applies to **b**, **d**, **f**), 20 µm in g, 40 µm in **h**

Rare CB-D28k-IR neurons were observed throughout the entire cranio-caudal extension of the calf LCN (a total of 40 clearly-visible neurons was counted) (Fig. 5a-f). The immunostaining was especially located in the somata and primary dendrites. The CB-D28k-IR neurons were mostly located at C1 level: C1 contained (28/40) 70% of neurons, C2 (4/40) 10%, and C3 (12/40) 30%. These neurons presented polygonal cell bodies with clearly-evident primary dendrites (Fig. 5g). The neuropil staining was mainly caused by the expression of an immunoreactivity in the fine dendritic branching. Generally, the neurons in the LCN were less intensely immunostained than those located in the superficial layers of the dorsal horn (Fig. 5h). The perikaryal areas of CB-D28K-IR neurons in the bovine LCN are reported in Table 7.

**Table 7:** Bovine lateral cervical nucleus: perikaryal area (µm^2^) of total neuronal population immunoreactive for calbindin-D28k

Spinal segments	Mean area ± standard deviation	Minimum area	Maximum area
C1	383.5 ± 95.7	251.9	559.8
C2	558.8 ± 197.9	261.2	560.6
C3	482.7 ± 179.7	355.6	609.7
Total C1-C3	420.9 ± 117.2	251.9	609.7

### Porcine lateral cervical nucleus: calbindin-D28k immunoreactive neurons

**Fig. 6:**
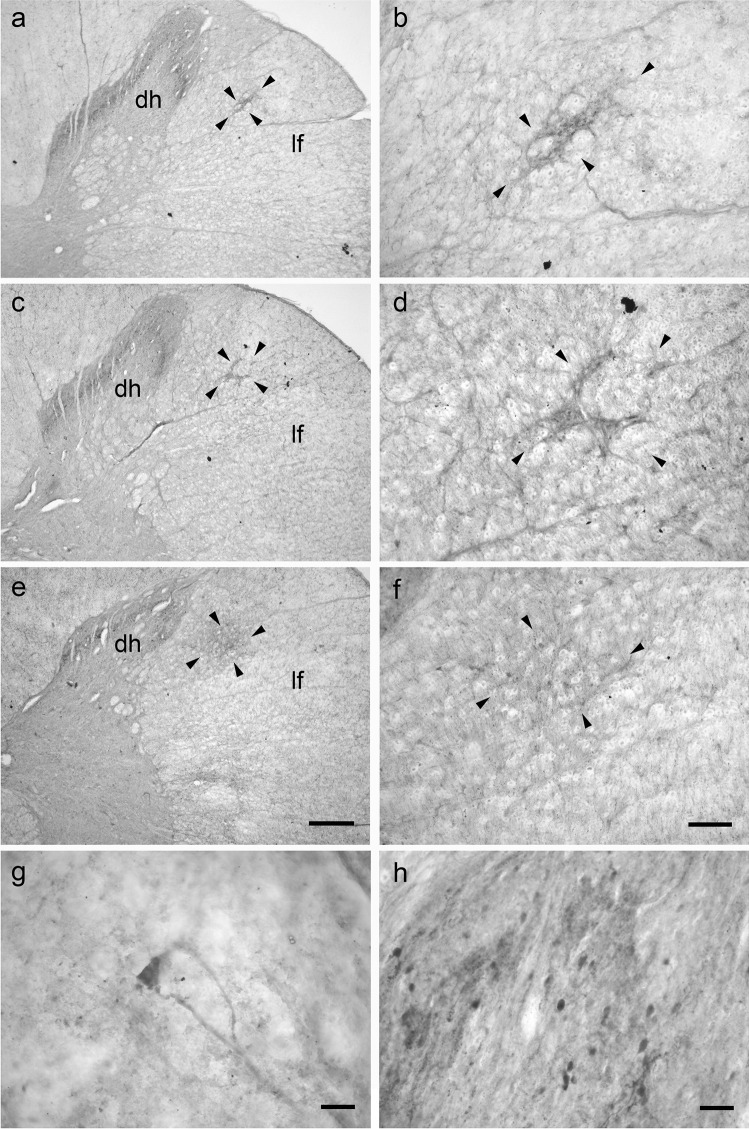
Brightfield photomicrographs of transverse sections showing the distribution of calbindin-D28K (CB-D28k) immunoreactivity in the porcine lateral cervical nucleus (bordered by arrowheads). The three sections are arranged from cranial to caudal direction: C1 (**a**-**b**), C2 (**c**-**d**) and C3 (**e**–**f**). CB-D28k-immunoreactive neurons are polygonal with evident primary dendrites (**g**). As in the bovine LCN, these neurons are more faintly immunostained than those of the superficial layers of the dorsal horn (**h**). Abbreviations: dh, dorsal horn; lf, lateral funiculus. Scale bar = 400 µm in e (applies to **a**, **c**, **e**) and 100 µm in f (applies to **b**, **d**, **f**), 20 µm in g, 40 µm in **h**

A moderate number (52) of CB-D28k-IR neurons was counted along the whole extension of the LCN (from C1 to C3) (Fig. 6a-f). These cells were usually polygonal with well-visible primary dendrites (Fig. 6g). As already seen in the calf, also the pig CB-D28k-IR neurons tended to decrease in a cranio-caudal direction: C1 contained (36/52) 69.2% neurons, C2 (8/52) 15.4%, and C3 (8/52) 15.4%. The LCN also contained fine dendritic processes that were immunoreactive for the CB-D28K. The neurons located in the superficial laminae of the dorsal horn were more intensely immunolabelled than those located in the LCN (Fig. 6h). The perikaryal areas of CB-D28k-IR neurons in the pig LCN are reported in Table 8.

**Table 8:** Swine lateral cervical nucleus: perikaryal area (µm^2^) of total neuronal population immunoreactive for calbindin-D28k

Spinal segments	Mean area ± standard deviation	Minimum area	Maximum area
C1	184.1 ± 45.1	130.4	246.7
C2	111.8 ± 29.2	91.1	132.4
C3	193.9 ± 66.7	146.7	241.1
Total C1-C3	174.4 ± 50.9	91.1	246.7

### Bovine and porcine lateral cervical nucleus: nNOS immunoreactivity

**Fig. 7:**
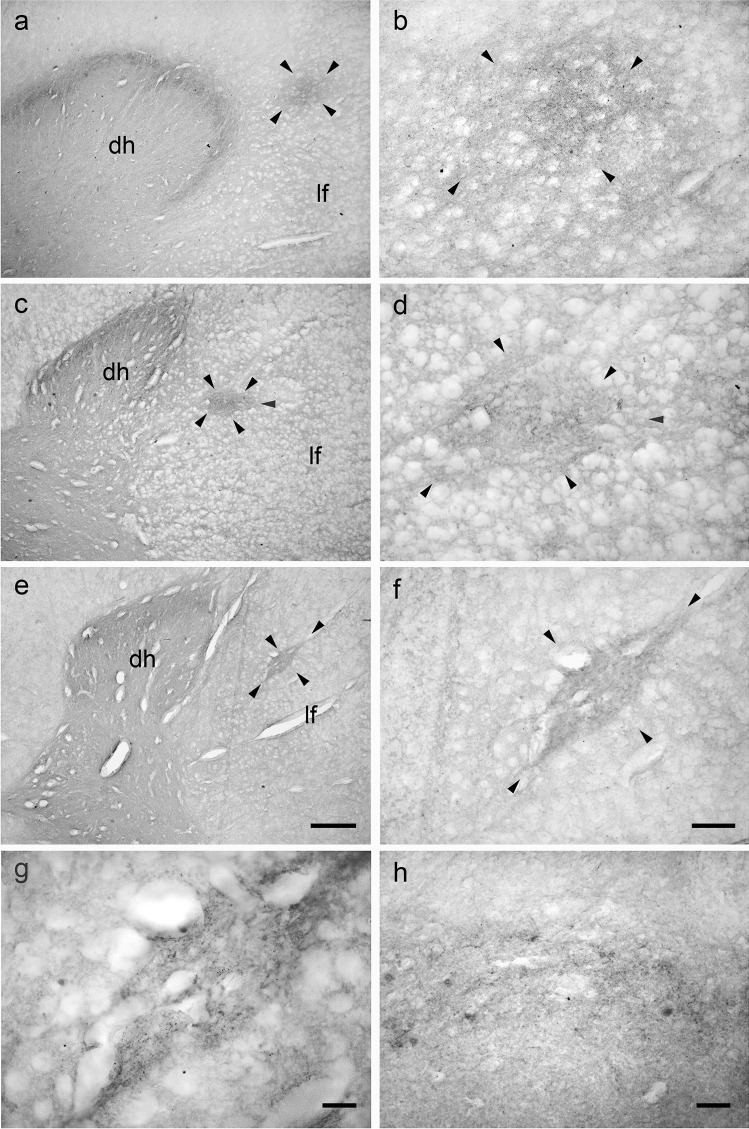
Brightfield photomicrographs of transverse sections showing the distribution of neuronal nitric oxide synthase (nNOS) immunoreactivity in the bovine lateral cervical nucleus (bordered by arrowheads). The three sections are arranged from cranial to caudal direction: C1 (a-b), C2 (c-d) and C3 (e–f). Note that the immunostaining is located only at neuropilar level (g). Superficial laminae of the dorsal horn are more intensely immunostained than LCN (h). Abbreviations: dh, dorsal horn; lf, lateral funiculus. Scale bar = 400 µm in e (applies to a, c, e) and 100 µm in f (applies to b, d, f), 40 µm in g, 40 µm in h

**Fig. 8:**
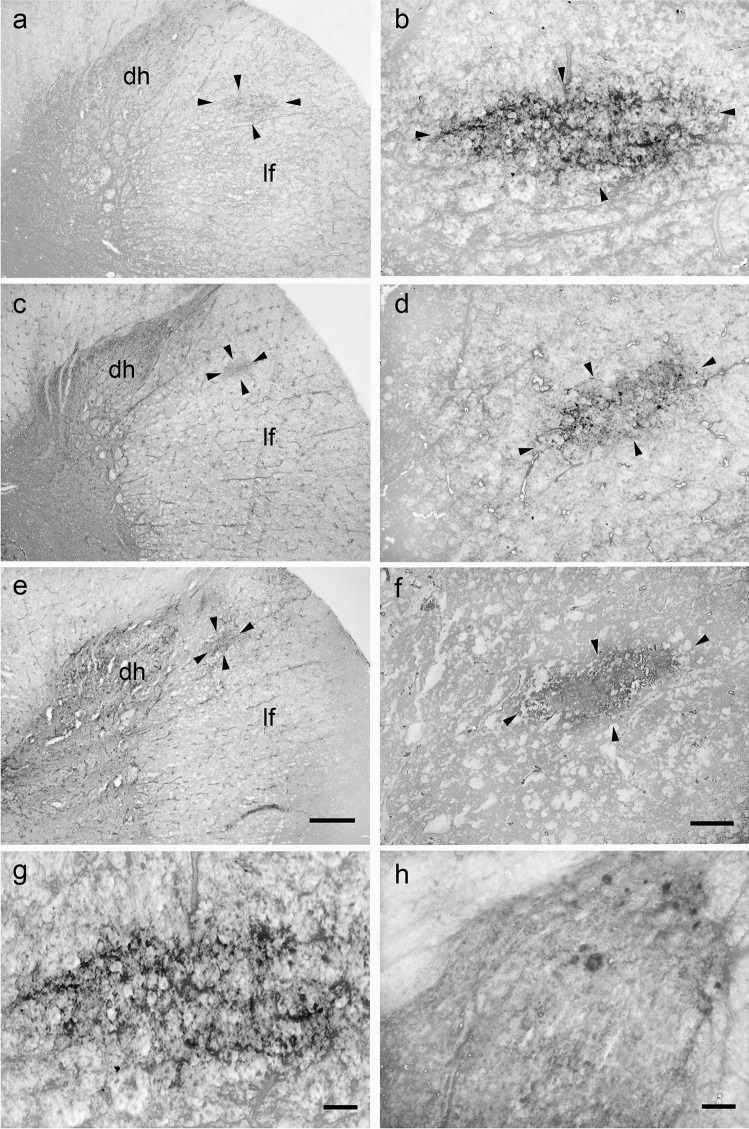
Brightfield photomicrographs of transverse sections showing the distribution of neuronal nitric oxide synthase (nNOS) immunoreactivity in the porcine lateral cervical nucleus (bordered by arrowheads). The three sections are arranged from cranial to caudal direction: C1 (**a**-**b**), C2 (**c**-**d**) and C3 (**e**–**f**). Only neuropilar elements show immunostaining (**g**). Laminae I-III of the dorsal horn are more intensely immunopositive than LCN (**h**). Abbreviations: dh, dorsal horn; lf, lateral funiculus. Scale bar = 400 µm in **e** (applies to **a**, **c**, **e**), 100 µm in **f** (applies to **b**, **d**, **f**), 40 µm in g and h

The immunoreactivity for nNOS was observed in the LCN (from C1 to C3) of both species (Figs. 7a-f and 8a-f). The staining mostly consisted of multiple varicose and diffuse neuropil staining, whereas no neurons were evidenced (Figs. 7g and 8g). In the laminae I-III of the dorsal horn, unlike what was observed in the LCN, immunoreactive somata were present (Figs. 7h and 8h).

## Discussion

Previous studies have examined the organization of the LCN in different mammalian species. These works have shown that carnivores show the most developed LCN (Giesler et al. 1987; Molander et al. 1989; Truex et al. 1970; Zhang et al. 2002). In the present study, the cytoarchitectonic organization of the bovine and porcine LCN was investigated by using a combination of thionine staining and morphometrical analyses. In addition, information regarding the distribution of the immunoreactivity for the CB-D28k and nNOS in the LCN of these species was provided. To estimate the calf and pig LCN thionine- or immunoperoxidase-stained neuronal count, we counted the neurons that contained a well-visible nucleus (as well as a nucleolus, only when analyzing thionine preparations). It should be emphasized that our primary objective was to describe the general organization of the calf and pig LCN, rather than calculate an accurate total neuronal count. Therefore, twelve nonconsecutive sections obtained from each cervical segment (of each animal) were analyzed, to obtain an anatomical characterization with general neuronal patterns without quantitative neuron counting. 

In agreement with previous investigations, the results of the present study show that the bovine and porcine LCN appear as a clearly defined column of gray matter located in the three cranial segments of the cervical spinal cord. Consistently with what had been reported for carnivores, most calf and pig LCN neurons were found to be located in the rostral part of the nucleus. In general, bovine and porcine LCN neurons are morphologically similar to the neurons observed in other species (Giesler et al. 1987; Molander et al. 1989; Truex et al. 1970; Zhang et al. 2002). Interestingly, the calf LCN showed a large variability in neuronal morphology, although the prevalent cell types were polygonal, analogously to what was observed in the pig. In addition, the size of LCN neurons was larger in calves than it was in pigs. Since previous studies have demonstrated that the neuronal size of the LCN neurons is not related to the animal body size (Truex et al. 1970), we could assume that the bovine LCN is more developed than that of the pig. This is the opposite of the situation found in other nuclei in the bovine compared to the pig (Graïc et al. 2018). Further studies on neuronal morphometry could clarify this point (Grisan et al. 2018; Corain et al. 2020). The bovine and porcine LCN contained a low percentage of CB-D28k-IR cells; these presented large somata in both species, suggesting their potential involvement in the cervicothalamic pathway. Indeed, neurons containing CB-D28k located in the spinal cord can emit long ascending projections to the supraspinal area. In addition, it was noted that the injection of horseradish peroxidase (HRP) in the cat thalamus resulted in the specific labelling of the large neurons located in the LNC (Craig and Burton 1979). The small CB-D28k-IR neurons of the LCN might be interpreted as local interneurons. Consistently, small-sized GABAergic neurons were observed in the monkey and cat LCN (Broman and Westman 1988; Broman and Blomqvist 1989a; Broman and Pubols 1996). The immunoreactivity for nNOS seems to be mainly expressed by thin processes that are present in the lateral cervical nucleus. Since the spinocervical tract originates from neurons located in the dorsal horn throughout the entire length of the spinal cord, the thin processes that immunoreacted to the nNOS could represent the terminal axonal portions of the numerous nNOS-IR found in laminae III e IV (Saito et al. 1994; Bombardi et al. 2013). In such a way, nitric oxide (NO) could modulate the synaptic activity of the glutamatergic spinocervical tracts (Broman et al. 1990). The somatosensory information, ultimately destined to the neocortex, is carried to the thalamic nuclei through three different main pathways: the dorsal column-medial lemniscus, the spinothalamic tract and the spinocervicothalamic pathway (Paxinos 2015). The present study, by reporting the presence of a well-developed LCN in bovines and, to some extent, in pigs, indicates that the spinocervicothalamic pathway conveys somatosensory and noxious information from the peripheral receptors to the higher centers of the brain in these species, as well. In the monkey, cat and rat, numerous substance P (SP)-immunoreactive fibers were also observed in the LCN (Giesler and Elde 1985; Broman and Blomqvist 1989b; Broman and Pubols 1993). Since SP immunoreactivity is associated with nociceptive function, our further studies will aim to determine the distribution of SP immunoreactivity in the LCN of Artiodactyls as well. By improving the knowledge regarding the nociceptive system of Artiodactyls, this study provides solid morphofunctional bases supporting the implementation of the welfare of animals, including food-producing animals.

## Data Availability

The datasets in this study are available from the corresponding author on reasonable request. All data and materials are available for publication.
